# ISMB 2005 Conference Report

**DOI:** 10.1371/journal.pcbi.0010033

**Published:** 2005-08-26

**Authors:** B. J Morrison McKay

The ISCB held its 13th annual Intelligent Systems for Molecular Biology Conference (ISMB) in Detroit, Michigan, June 25–29, 2005. Considering this conference has moved to many points on the globe in its 13 years, this location was not so far away geographically from the first ISMB held in Bethesda, Maryland, in 1993, but the increase in numbers in all categories—attendance, presentations, and special intrest group meetings and tutorials—put it in a separate stratosphere altogether (see Table 1).

**Table 1:**
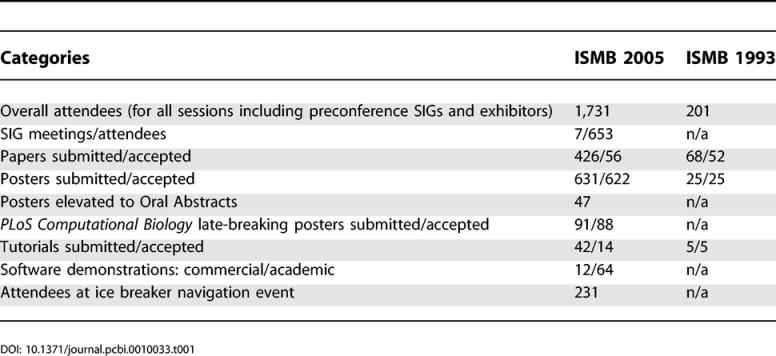
ISMB Growth since Inception

ISMB/ECCB 2004, held in Scotland last year as a joint conference with the European Conference on Computational Biology (ECCB), resulted in the highest attendance of any ISMB or any ECCB on record, with 2,136 total attendees. Given a variety of challenges, and given that the meeting in Detroit was not co-located with ECCB or any other conference in our field, ISMB 2005 drew attendees surprisingly well. In fact, ISMB 2005 had the second-highest attendance ever, and was the most highly attended North American ISMB to date.

As in years past, seven special interest group (SIG) meetings preceded this year's conference, enabling registrants to come for both the SIGs and the main conference, or attend just the SIGs. The SIGs are fast becoming one of the main attractions of ISMB, with topics as varied as the “Bioinformatics Open Source Conference,” “Alternative Splicing,” and “Bioinformatics and Disease.” Some 653 delegates arrived in Detroit early for these one- or two- day focused sessions—485 of whom then stayed on for ISMB. The “Alternative Splicing” SIG had the highest number of attendees, but the beauty of this year's SIG arrangements was the ability for all registrants to move among SIGs to capture more of a knowledge base on the varied topics. If you'd like to view the full list of options that were available this year, the preconference SIG abstracts are available at http://www.iscb.org/ismb2005/sigs.html.

Also serving as a precursor to the main conference were 14 half-day tutorial sessions that were attended by 548 delegates. New and interesting tutorials are sought for each ISMB, and this year's tutorial abstracts on the conference Web site (http://www.iscb.org/ismb2005/tutorials.html) include a link to the original proposal selected for one of the limited tutorial slots. If you are interested in presenting a tutorial proposal for 2006, we encourage you to follow the links at http://www.iscb.org/ismb2005/tutorials.html to gather ideas on preparation and submission of tutorials. Then watch for the call for tutorials via E-mail and on the ISMB 2006 Web site.

A very special few were recognized for their outstanding papers with the GlaxoSmithKline Bioinformatics Prizes for best paper. ISCB congratulates the winners (see Box 1). ISCB also bestowed its two highest awards of recognition during the conference. The Overton Prize, for a scientist in the early-to-mid-career stage, went to Ewan Birney, and the Senior Scientist Accomplishment Award went to Janet Thornton, both from the European Bioinformatics Institute (EBI). This was the first time both awards had gone to scientists at the same institution—a remarkable statement about the quality of science being generated from the EBI. Both award winners gave keynote lectures on the final day of ISMB—a very fitting way to end a successful conference.

Please commit to joining us for ISMB 2006 in Fortaleza, Cearà, Brazil, August 6–10 (http://www.iscb.org/ismb2006). Program co-chairs Phil Bourne, Editor-in-Chief of *PLoS Computational Biology* and Professor of Pharmacology at University of California at San Diego, and Søren Brunak, Director of the Center for Biological Sequence Analysis of the Technical University of Denmark, are working together with conference chair Goran Neshich of Empresa Brasileira de Pesquisa Agropecuária to develop one of the most interesting and innovative ISMBs yet. Additionally, the 20th Anniversary Swiss-Prot conference is co-locating to take place just prior to ISMB, and several Nobel laureates have already committed to speaking at each conference. Brazil has a strong lure, with its endless coastline of white-sand beaches, but the lure will be all about the science as we prepare to roll out an exceptional ISMB in the coming year. As 97% of this year's postconference-survey respondents indicated they will attend an ISMB again, we're counting on seeing you in Fortaleza! 

##

**Box 1. GlaxoSmithKline Bioinformatics Winners**

**Best Papers:**

**Matthew Dimmic** Dimmic MW, Hubisz MJ, Bustamante CD, Nielsen R (2005) Detecting coevolving amino acid sites using bayesian mutational mapping. Bioinformatics 21: il26–il35.

**John Spouge**

Tharakaraman K, Mariño-Ramirez L, Sheetlin S, Landsman DL, Spouge J (2005) Genomic landmarks can aid in the identification of regulatory elements. Bioinformatics 21: i440–i448.

**Best Student Papers:**

**Elena Nabieva** Nabieva E, Jim K, Agarwal A, Chazelle B, Singh M (2005) Whole-proteome prediction of protein function via graph-theoretic analysis of interaction maps. Bioinformatics 21: i302–i310.

**Alena Shmygelska** Shmygelska A (2005) Search for folding nuclei in native protein structures. Bioinformatics 21: i394–i402.

**Honorable Mention Paper:**

**Charlotte Deane** Winstanley WF, Abeln S, Deane CM (2005) How old is your fold? Bioinformatics 21: i449–i458.

**Honorable Mention Student Paper:**

**Shaun Mahony** Mahony S, Golden A, Smith TJ, Benos PV (2005) Improved detection of DNA motifs using a self-organized clustering of familial binding profiles. Bioinformatics 21: i283–i291.

